# Optimization of Almond Beverage Enriched with Omega-3 Fatty Acids by Adding Brown Flaxseeds (*Linum usitatissimum* L.) Using D-Optimal Mixing Diagram Method

**DOI:** 10.17113/ftb.63.02.25.8460

**Published:** 2025-06

**Authors:** Zeinab El Hajj Hussein, Jiuliane Martins Silva, Matheus Campos Castro, Nathalia Elias Borges, Bruno Henrique Figueiredo Saqueti

**Affiliations:** 1 Department of Food Science, State University of Maringá, Avenue Colombo, 5790, University Campus, 87020-900, Maringá, Paraná, Brazil; 2 Department of Chemistry, State University of Maringá, Avenue Colombo, 5790, University Campus, 87020-900, Maringá, Paraná, Brazil

**Keywords:** response surface methodology, optimization, almond drink, flaxseed, omega-3 fatty acids

## Abstract

**Research background:**

The almond beverage enriched with flaxseed is an important source of α-linolenic acid (ALA), an essential omega-3 fatty acid that the human body cannot synthesize and must obtain it through the diet. Although omega-3 fatty acids are essential for all people, this beverage is particularly beneficial for those who do not consume fish, such as vegans and vegetarians, as it is a plant-based source of ALA. Its versatility allows it to be easily incorporated into different recipes and daily meals. Therefore, this study aims to optimize a plant-based almond beverage enriched with flaxseed, offering a natural and completely vegan source of omega-3 fatty acids.

**Experimental approach:**

The initial formulation of the drink comprised 75 % raw almonds and 25 % flaxseed mixture, water (in 1:6 ratio) and 4 % sweetener. Following preliminary tests, the beverage was optimized using the mixing method to determine the ideal proportions of the ingredients. This process resulted in 16 samples, each with different minimum and maximum amounts of each ingredient, which were replicated and analyzed. One of the formulations achieved an optimal mass fraction of 4.27 mg/g of omega-3 fatty acids and a viscosity of 6.03 mPa^.^s. The physicochemical properties, bioactive compounds, fatty acid composition and lipid profile of the optimized beverage were evaluated.

**Results and conclusions:**

The addition of flaxseed had a significant effect on the lipid profile and increased the alpha-linolenic acid content in the beverage by 1960 times, eventually reaching 3.92 %. This optimization enriched the beverage with omega-3 fatty acids and improved the concentration of antioxidants and carotenoids. Importantly, these improvements did not significantly affect the color and viscosity of the final product, creating an affordable option that can benefit the vegan and vegetarian community.

**Novelty and scientific contribution:**

This study shows that the response surface model effectively identified the ideal composition for the beverage, leading to an optimized formulation of a plant-based drink. This composition could have promising applications in the food industry.

## INTRODUCTION

Plant-based diets have become a trend in recent years due to their environmental and health effects (*1*). These diets include different patterns, such as veganism, which involves the complete elimination of animal products, and lacto-ovo vegetarianism, which excludes meat and fish but includes dairy products and eggs. Lacto-ovo vegetarianism can be further divided into lacto-vegetarian (which includes dairy products but not eggs) and ovo-vegetarian (which includes eggs but not dairy products) diets (*2*).

With the spread of veganism, speciesism, *i.e.* discrimination against animals based on their species, is on the decline. In Brazil, 14 % of the population declare themselves vegetarian, according to a survey conducted by IBOPE in April 2018. In the metropolitan regions of São Paulo, Curitiba, Recife, and Rio de Janeiro, this figure rises to 16 %. The statistics showed a 75 % increase in 2012, when the same survey showed that the percentage of the Brazilian population in the metropolitan regions declared themselves vegetarian was 8 %. In 2018, almost 30 million Brazilians claimed to be vegetarian – a number greater than the population of all of Australia and New Zealand combined (*3*).

Although vegetarian diets are associated with several health benefits, there are significant concerns, especially when the diet is poorly planned (*4*). These concerns may arise from inadequate varieties and quantities of foods consumed, which can predispose individuals to nutritional deficiencies, such as vitamins B12, D and A, minerals such as calcium, iron, zinc and selenium, as well as iodine, protein and omega-3 fatty acids (*5*).

Adopting a vegetarian diet causes a deficiency of omega-3 fatty acids, especially eicosapentaenoic acid (EPA) and docosahexaenoic acid (DHA) found in cold-water fish, due to their absence in the diet (*6*). Alpha-linolenic acid (ALA) is the leading omega-3 fatty acid in vegetarian diet. Since it is a forerunner of EPA and DHA, its consumption is necessary because it is an essential fatty acid, not synthesized by the human body, and an indispensable component for a healthy and balanced diet (*7*).

Walnut, flaxseed, chia seed, canola, hemp, echium and perilla seeds are plant-based sources of ALA, which have been associated with many health benefits, including anti-inflammatory, antibacterial, antidiabetic, antioxidant, antihypertensive and neuroprotective activity. When included in a balanced diet, ALA may also have a beneficial effect on body mass reduction (*8*).

Flaxseed, also known as linseed (*Linum usitatissimum* L. from the Linaceae family) or linseed oil, is the best source of omega-3 fatty acids in the form of ALA (*9*). The 18:3n-3 in flaxseed corresponds to 55 %, more than half of its overall fat content, which is a higher percentage than other vegetable sources of ALA (*10*). ALA can be converted to long-chain polyunsaturated fatty acids (EPA and DHA) by gradual desaturation and chain elongation in the presence of desaturase enzymes. Its conversion efficiency is minimal (<8 % of ALA to EPA and <4 % of ALA to DHA) because it competes with linoleic acid for the same conversion pathway. Nevertheless, its role in long-term dietary intake remains significant (*11*).

Foods and beverages fortified with plant-based omega-3 fatty acids are gaining attention from the industry as they appeal to consumers who follow a plant-based diet and seek delicious drinks with natural, healthy and nutritious ingredients (*12*). The growth of this audience poses new challenges for the food industry and opens up new market possibilities. The global sector of dairy alternatives was estimated at USD 27 billion in 2023 and is expected to grow at a compound annual growth rate of 10.1 %, reaching USD 43.6 billion by 2028 (*13*).

The plant-based almond drink is a product intended for consumption by people who follow a plant-based diet. It is a colloidal dispersion obtained after processing almonds with water, filtering and homogenizing the resulting milky white liquid (*14*).

A mass of 100 g of plant-based almond drink contains lipids (1.99 g), proteins (0.47 g), carbohydrates (6.02 g) and minerals such as calcium, potassium, magnesium and phosphorus (*15*), with a lower number of calories, ranging from 45 to 56 kcal. The drink is deficient in omega-3 fatty acids, because almonds contain an insignificant amount of α-linolenic acid (C18:3n3) (*16*), but are rich in oleic acid (C18:1n−9; 63 %) and linoleic acid (C18:2n-6; 24 %) (*17*).

In recent years, flaxseed has become increasingly popular as a key ingredient in functional food products such as bread, dairy products, beverages and ready-to-eat meals due to its high nutritional value and numerous health benefits (*18*). Several studies have addressed the fortification of products with flaxseed-derived omega-3 fatty acids, such as dairy milk fortified with flaxseed oil (*19*), yogurt incorporated with flaxseed mucilage and oil-free or encapsulated flaxseed (*20*). Encapsulated and co-encapsulated flaxseed oil in hydrogel spheres composed of calcium alginate and carboxymethyl cellulose (CA-CMC), produced by the extrusion dripping technique, was used for the development of functional orange juice (*21*). These studies concluded that flaxseed successfully serves as a potential delivery system for omega-3 fatty acids in the form of ALA.

To the best of our knowledge, there is a lack of studies investigating the enrichment of almond-based plant beverages with omega-3 fatty acids by the addition of flaxseed during processing. In this context, the present study aims to develop an almond-based plant beverage enriched with flaxseed, using mixture design methodologies to optimize the proportions of ingredients. Additionally, multivariate statistical techniques will be used to evaluate the effects of the addition of flaxseed on the lipid profile, fatty acid composition and physicochemical properties of the beverage. These approaches will ensure a comprehensive understanding of the formulation and its nutritional improvements.

## MATERIALS AND METHODS

### Materials and reagents

Sodium hydroxide, methanol, sulfuric acid, hexane, acetone, isopropyl alcohol and sodium sulfate were purchased from Synth Lab (São Paulo, Brazil). The standards methyl tricosanoic acid (23:0), analytical standard mixture of fatty acid methyl esters (FAMEs C4-C24), 2,2-diphenyl-1-picrylhydrazyl (DPPH) and 6-hydroxy-2,5,7,8-tetramethylchroman-2-carboxylic acid (Trolox) were purchased from MilliporeSigma, Merck (St Louis, MO, USA).

The ingredients required for the preparation of the drink were obtained from the local market in Maringá, Paraná, Brazil. These included 1000 g of raw almond (*Prunus dulcis*) seeds, 500 g of brown flaxseed (*Linum usitatissimum* L.), 300 g of xylitol and 300 g of erythritol. All ingredients were obtained from a unique batch from Terra Verde Company in Maringá, Paraná. They were then transported to the Laboratory of Applied Analytics for Lipids, Sterols, and Antioxidants at the State University of Maringá (APLE-A, UEM) for further testing.

### Preliminary test

To prepare an almond drink fortified with omega-3 fatty acids and determine the standard formulation, an already commercialized almond drink available for consumption was used as a reference. Preference was given to a product with natural ingredients without colorings and preservatives. The almond drink of Iracema (Dobrada, São Paulo, Brazil) brand, consisting of water, almonds, natural flavors and natural sweeteners erythritol and stevia, was chosen because its composition is similar to that of the prepared sample.

First, a preliminary test was carried out to determine the amount of flaxseed required to increase the omega-3 content of the drink without altering its flavor and viscosity. After the test, the following formulation was selected: 75 % almond seeds and 25 % flaxseeds mixed with water in 1:6 ratio and 4 % xylitol and erythritol.

The sample was prepared according to Manzoor *et al.* (*22*) and then the omega-3 fatty acid content of the sample was analyzed by gas chromatography (model 7890B; Agilent Technologies, Santa Clara, CA, USA). The results showed that the addition of 25 % flaxseed to the drink increased the omega-3 content of the sample, a value that is consistent with the data in Regulatory Instruction No. 28 of 26 July 2018 of the National Health Surveillance Agency (ANVISA) (*23*). This regulation establishes minimum levels of nutrients, bioactive substances, enzymes and probiotics that must be provided in foods according to the daily consumption recommendations and the population group indicated by the manufacturer. According to this regulation, a food intended to be a source of alpha-linolenic acid must contain between 0.24 and 2.4 g per serving to meet the daily requirements of people aged nine and older. Additionally, the Resolution of the Collegiate Board No. 54 of 12 November 2012 of ANVISA (*24*), which contains the technical regulation on supplementary nutritional information, establishes a minimum content of 300 mg of alpha-linolenic acid per 100 mL in prepared foods in order for them to be considered a source.

Therefore, the vegetable drink made with 25 % flaxseed can be recognized as fortified, enriched or a source of omega-3 fatty acids.

### Preparation of almond and flaxseed drink

The vegetable drink containing almonds and flaxseeds was prepared following the method described by Manzoor *et al*. (*22*), with some modifications. First, (100.000±0.001) g of almond seeds was hydrated in distilled water at (4.0±0.0) °C in a ratio of 1:3 (*m*/*V*) for 16–19 h. After soaking, the seeds were drained, washed and weighed again to determine the amount of the absorbed water. Of the 100 g almond seeds, 25 % (25 g) were replaced with flaxseeds, while 75 % (75 g) remained almond seeds.

The seeds were mixed with distilled water in a ratio of 1:6 (*m*/*V*) and ground in a blender (Walita Series 5000 RI2240/9-1200 W; Philips, São Paulo, Brazil) for 150 s. The beverage was filtered through a cloth strainer, then 4 % (4 g) of a mixture of xylitol and erythritol was added to the obtained liquid and homogenized in the blender for 2 min.

The beverage was then bottled in glass bottles, previously sterilized with hot water steam at 100 °C for 10 min, followed by pasteurization at 75 °C for 15 s in a water bath, according to the method of Nagarajappa and Battula (*25*). The temperature was monitored during the process with a digital thermometer and samples were then rapidly cooled in an ice bath at 4–5 °C. Thus obtained beverage was stored under refrigeration at 4 °C and later used for the analysis.

### Experimental design using the D-optimal mixing method

The mixing method numerical and graphical optimization techniques were used to optimize the composition of the ingredients of the almond-based vegetable drink. The aim was to determine the ideal amount of almonds, flaxseed and sweeteners to produce a beverage enriched with omega-3 fatty acids while maintaining the desired viscosity, taking into account the gelling properties of flaxseed after hydration. A D-optimal mixture design was developed using Design-Expert® Software, v. 7.0.0 (*26*).

The preliminary test showed that the amount of flaxseed of 25 % was ideal for the preparation of the drink with an increased omega-3 content. A mixture diagram was used to optimize the preparation and provide information on the effect of each independent variable in the preparation of the drink. The extraction conditions according to the parameters and levels for D-optimal design are minimum mass of 15, 5 and 2 g and maximum mass of 17, 6 and 5 g of almonds, flaxseeds and sweetener, respectively.

**Fig. S1:**
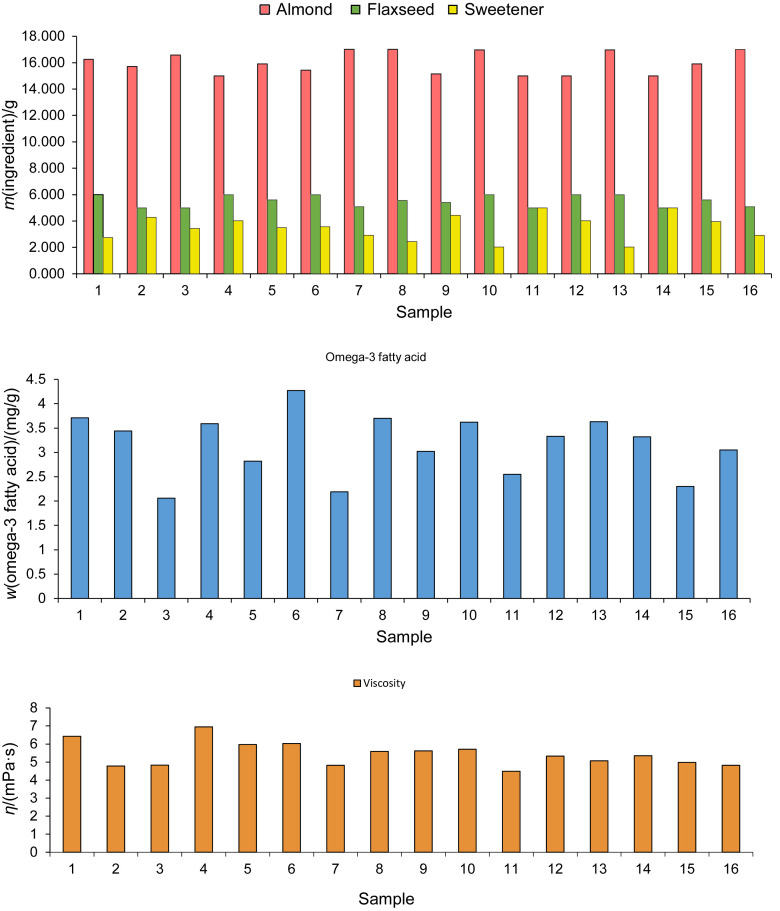
Experimental results from the D-optimal design and the respective responses: a) the independent variables of the samples analyzed, b) response 1 (omega-3) and c) response 2 (viscosity)

Sixteen samples were analyzed using the central composite face-centered design, with three independent variables and two responses. The experimental design followed the central composite design matrix in standardized order (Table 1), with the conditions indicated in Fig. S1.

**Table 1:** Experimental results from the D-optimal design and the respective responses

**Sample**	Independent variable			Response	
	*m*(almond)/g	*m*(flaxseed)/g	*m*(sweetener)/g	*w*(omega-3 fatty acid)/ (mg/g)	*η*/(mPa·s)
**1**	16.254	6.000	2.746	3.71	6.43
**2**	15.714	5.000	4.286	3.44	4.78
**3**	16.568	5.000	3.432	2.06	4.83
**4**	15.000	5.984	4.016	3.59	6.95
**5**	15.906	5.600	3.494	2.82	5.97
**6**	15.426	6.000	3.571	4.27	6.03
**7**	17.000	5.082	2.918	2.19	4.82
**8**	17.000	5.562	2.438	3.70	5.59
**9**	15.154	5.417	4.428	3.02	5.62
**10**	16.961	6.000	2.039	3.62	5.71
**11**	15.000	5.001	4.999	2.55	4.49
**12**	15.000	5.984	4.016	3.33	5.33
**13**	16.961	6.000	2.039	3.63	5.07
**14**	15.000	5.001	4.999	3.32	5.35
**15**	15.906	5.600	3.949	2.30	4.98
**16**	17.000	5.082	2.918	3.05	4.82

### Physicochemical properties

The viscosity of the almond drink was measured at 25 °C using a HAAKE MARS controlled stress rheometer (Thermo Fisher Scientific Inc., Waltham, MA, USA), equipped with a cone-and-plate geometry (35 mm diameter, fixed gap of 0.052 mm). The samples were carefully placed on the lower plate and allowed to return to equilibrium for at least 1 min before starting the analysis. A gradient range from 0 to 500 was used.

Titratable acidity was determined according to AOAC method 947.05 (*27*). A volume of 10 mL of the sample was transferred to a 125-mL Erlenmeyer flask, and 10 mL of water and 4 drops of the acid-base indicator 1 % (*m*/*V*) phenolphthalein were added. The sample was titrated using a standardized 0.1 mol/L sodium hydroxide solution and shaken until a permanent pink color was observed for 30 s. The results were expressed as a percentage of lactic acid.

To determine the ash content, the AOAC method 942.05 was used (*28*), in which 10 g of the sample is incinerated in a porcelain crucible in a muffle furnace at a controlled temperature of 600 °C, resulting in the evaporation of water, volatile substances and oxidation of organic matter.

Protein content was determined using AOAC method 990.03 (*29*). This method consists of three main steps: (*i*) sample digestion, (*ii*) distillation, and (*iii*) titration. Protein content is determined based on nitrogen content, where nitrogen normally accounts for 16 % of the mass of the protein sample. Carbohydrate content was calculated by subtracting the sum of the mass fractions (in %) of other components such as water, protein, fat and ash from 100 (*30*).

The total caloric value was determined using the Atwater conversion values ​​of 4.07 kcal per g of protein, 3.47 kcal per g of carbohydrates and 8.37 kcal per g of lipids (*31*). The pH was determined using a benchtop pH meter with a combined glass electrode (PHS3BW; Bel Engineering, Monza, Italy). The pH meter was calibrated with standard pH=4.0 and 9.0 at 20 °C prior to sample analysis. Soluble solids were determined by refractometry, using a portable refractometer (model RTA-100; Abbe, São Paulo, Brazil) with a °Brix scale of 0–30 (*32*). Humidity was measured on an infrared scale (model i-Thermo 163m; Bel Engineering). The analysis was performed in triplicate, using approx. 0.500 g of sample in each run.

The color parameters were analyzed using a digital colorimeter CR-400 (Konica Minolta Sensing Americas Inc., São Paulo, Brazil). Measurements were taken at three points on each side of the plant-based drinks and the results were expressed as L* (luminosity), a* (variation from red to green) and b* (variation from yellow to blue) according to the CIE system (*33*).

### Lipid extraction

Lipids were extracted from the samples according to the method developed by Bligh and Dyer (*34*), which was carried out in triplicate. In the first step, approx. 100 g of the sample were weighed and 100 mL of chloroform and 200 mL of methanol were added. The mixture was then homogenized for 2 min in a model 653 magnetic stirrer (Fisatom, São Paulo, Brazil). Next, 100 mL of chloroform were added and the mixture was stirred again for 30 s. Another 100 mL of water were added to the mixture to separate the two phases. The mixture was stirred for another 30 s. The sample was filtered with Whatman filter paper no. 1 (Pró Analysis, Porto Alegre, Brazil) in a Büchner funnel under vacuum and the filtrate was transferred to a separatory funnel. After separation, the organic (lower) phase was collected in a 250-mL flat-bottom flask (Duran Glass-Schott, Barcelona, ​​Germany) and the solvent was evaporated in a rotary evaporator model 802 (Fisatom). The lipid samples were collected and kept in a freezer at −18 °C until analysis.

### Direct derivatization

Direct derivatization was carried out according to the method proposed by Sinosaki *et al.* (*35*). To the vegetable beverage sample (100 mg), 2.0 mL of 1.25 mol/L NaOH in methanol were added, the mixture was homogenized and placed in an Elmasonic P ultrasonic bath (Elma, São Paulo, Brazil) at a frequency of 37 kHz for 5 min. Subsequently, 2.0 mL of 1.5 mol/L H_2_SO_4_ in methanol were added and the mixture was placed in the ultrasonic bath for 5 min. Finally, 1 mL of hexane was added, stirred for 30 h in a vortex tube shaker at 2800 rpm (Kasvi Laboratory, Londrina, Paraná, Brazil) and centrifuged for 1 min at approx. 3.57×*g* (Kasvi Laboratory). Finally, 500 µL of internal standard (23:0) were added. The supernatant was then collected and injected into the gas chromatograph-flame ionization detector (GC-FID) (model Agilent 7890B; Agilent Technologies).

### Fatty acid determination by GC-FID

Fatty acid methyl esters (FAMEs) were separated in the GC-2010 Plus gas chromatograph (Shimadzu, São Paulo, Brazil) equipped with a flame ionization detector (FID), a split/splitless injector and a CP-7420 fused silica capillary column (selected FAME, 100.0 mm long, 0.25 mm inner diameter and 0.25 µm cyanopropyl film as stationary phase). The gas flows were 1.2 mL/min for carrier gas (H_2_), 30.0 mL/min for make-up gas (N_2_) and in the FID 35.0 and 350.0 mL/min for gas (H_2_) and synthetic air, respectively. The samples were injected in split mode, at a ratio of 1:20 and the injection volume was 1.0 µL. The column was heated with a heating ramp, starting at 65 °C for 4 min, then to 185 °C at 16 °C/min and held for 12 min, and finally heated to 235 °C at 20 °C/min and held for 9 min, with a total time of 35 min. The FAMEs were identified by comparing the retention times of the samples with the analytical standards of FAMEs (C4-C24). Theoretical FID correction values were applied to obtain the fatty acid mass fractions according to Visentainer (*36*), using the following equation:


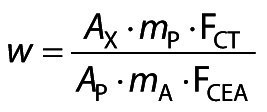
 /1/

where *w* is the mass fraction of the fatty acid (mg/g of sample), *A*_X_ is the peak area of the fatty acids, *A*_p_ is the peak area of the internal standard (23:0), *m*_p_ is the mass of internal standard (23:0) added to the sample (mg), *m*_A_ is the mass of the sample (g), F_CT_ is the theoretical correction factor of the flame ionization detector (FID) and F_CEA_ is the conversion factor from methyl ester to fatty acid.

### DPPH assay

The antioxidant activity, *i.e.* the 2,2-diphenyl-1-picrylhydrazyl (DPPH) free radical scavenging method, was performed according to the method described by Rodrigues *et al.* (*37*). To perform the antioxidant assay, the beverage extracts were previously prepared, 2.5 g of samples were weighed and 4.0 mL of 50 % methanol were added. This solution was then vortexed for 1 min and left to stand at room temperature (25 °C) for 60 min. It was then centrifuged at approx. 13.83×*g* for 25 min using an Eppendorf 5804 centrifuge (Eppendorf, Hamburg, Germany). The supernatant was collected and transferred to a 10-mL volumetric flask. A volume of 4 mL of 70 % acetone was added to the residue from the first extraction, vortexed for 1 min and allowed to stand at room temperature (25 °C) for 60 min. It was then centrifuged at approx. 13.83×*g* for 25 min using an Eppendorf 5804 centrifuge (Eppendorf), the supernatant was added to the other supernatant in the 10-mL volumetric flask and finally the volume of the flask was made up with distilled water. The beverage extracts (25 µL) were added to 2.0 mL of the methanolic DPPH solution (6 µg/250 mL). The mixture was then gently shaken and kept at room temperature (25 °C) for 30 min in the absence of light. The absorbance was measured at 517 nm using a UV-VIS spectrophotometer (Genesys 10S; Thermo Scientific, Thermo Fisher Scientific). A standard curve with Trolox (0–0.3 mg/mL) was used to calculate the antioxidant activity and the result was expressed in μmol/g of sample.

### Carotenoid content determination

Carotenoid content was determined by the method of Higby (*38*). The carotenoids were extracted with hexane and isopropyl alcohol was added to obtain a single phase. First, 5 g of the sample were weighed in an amber Erlenmeyer flask and 15 mL of isopropyl alcohol and 5 mL of hexane were added. This solution was then stirred for 1 min and then transferred to a 125-mL separatory funnel covered with aluminium foil, its volume was made up with water and left to rest for 30 min. After this time, the the aqueous phase was removed, leaving only the yellow phase. After extracting three times with water (each time resting for 30 min), the contents of the funnel were filtered through cotton sprayed with anhydrous sodium sulfate and the filtrate was collected in a 25-mL amber volumetric flask. Subsequently, 2.5 mL of acetone were added to the flask and the volume was made up with hexane. The absorbance was measured at 450 nm using a UV-VIS spectrophotometer (Genesys 10S; Thermo Scientific, Thermo Fisher Scientific). The carotenoid mass fraction was expressed in μg/100 g of sample and determined according to the following equation:


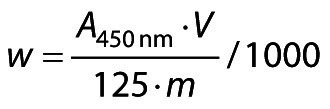
 /2/

where *A*_450 nm_ is the absorbance value of the beverages, *V* is the volume of the sample used (mL), *m* is the mass of the sample (g) and 125 is a factor for quantification of carotenoids.

### Statistical analysis

The experiment followed a completely randomized design and the analyses were performed in triplicate. Data were subjected to Analysis of Variance (ANOVA) and Tukey's multiple comparison test (p<0.05) using XLSTAT software (*39*).

## RESULTS AND DISCUSSION

### Variability, optimization and validation by D-optimal mixing method

**Fig. S2:**
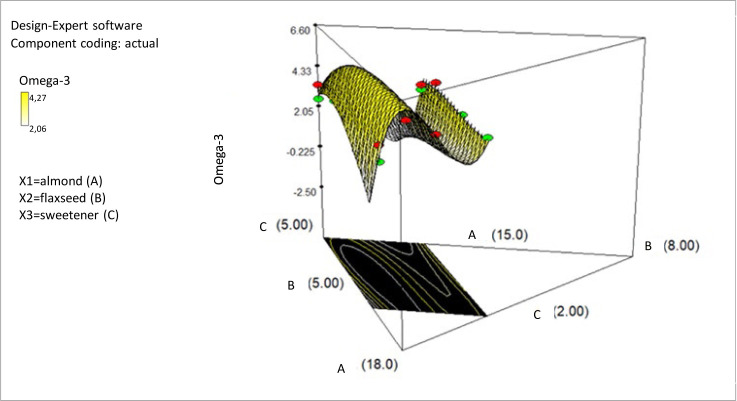
Response surface plot for omega-3 optimization

The almond-based vegetable drink enriched with plant-based omega-3 fatty acids from flaxseed is an ideal option to meet the nutritional needs for omega-3 fatty acids of vegans and to integrate it into a healthy and balanced diet. In the present study, numerical optimization of multiple responses was used to identify the optimal formulation within the overall optimization, which contains 15.426 g almond seeds, 6 g flaxseed and 3.571 g xylitol and erythritol and achieved the highest omega-3 fatty acid mass fraction of 4.27 mg/g. In contrast, the formulation with 16.568 g almond seeds, 5 g flaxseed and 3.432 g xylitol and erythritol resulted in the lowest omega-3 fatty acid mass fraction of 2.06 mg/g (Table 1). The 3D surface plot that shows the maximum desirability selected under the optimal conditions shows a highest omega-3 fatty acid mass fraction of 4.27 mg/g (Fig. S2).

**Fig. S3:**
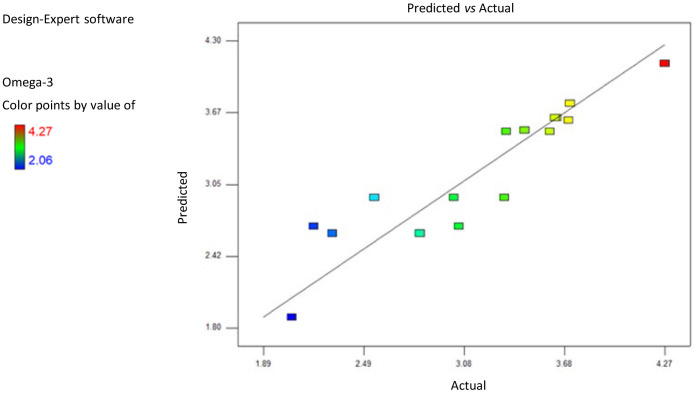
Graph of the predicted *versus* actual values of omega-3 fatty acids

Using the modified quadratic model, the experimental and predicted mass fractions of omega-3 fatty acids are shown in Fig. S3. The close proximity of the model prediction to the trial dice can be observed, which confirms the validity of the regression model.

**Fig. S4:**
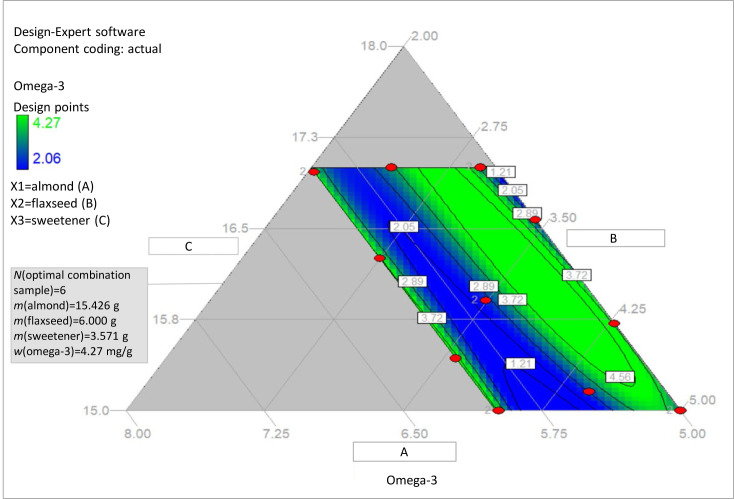
Triangle graph for mixture optimization

The optimization process was verified using the graphical optimization technique (Fig. S4), where the triangle graph for mixture optimization was generated to identify the optimal regions of the omega-3-rich formulation. According to the graph, all three variables significantly affected the omega-3 fatty acid mass fraction. The omega-3 fatty acid mass fraction was lowest at the medium mass of flaxseed and increased as the mass of flaxseed increased. The green area in the triangle graph represents an optimal combination of the ingredients for the beverage with the highest mass fraction of omega-3 fatty acids (almost 100 %), while the blue area represents the lowest composition.

Numerical optimization was carried out to determine the optimal ingredients for the production of the almond drink fortified with omega-3 fatty acid-rich flaxseed using the analysis of variance (ANOVA) test. The experiments were done in triplicate to ensure the accuracy of the model (Table 2).

**Table 2:** Numerical optimization of the results obtained using ANOVA

Sample	Independent variable			Predicted	Response
	*m*(almond)/g	*m*(flaxseed)/g	*m*(sweetener)/g	*w*(omega-3 fatty acid)/(mg/g)	*w*(omega-3 fatty acid)/(mg/g)
1	15.744	5.103	4.153	4.03	4.22
2	17.000	5.254	2.746	4.35	4.96
3	16.017	5.197	3.876	3.98	3.52

**Fig. S5:**
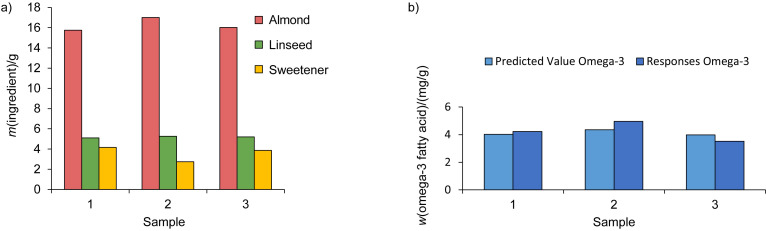
Numerical optimization of the results obtained using ANOVA: a) the independent variables of the samples analyzed, and b) the numerical optimization of omega-3

As shown in Table 2, the values ​​predicted by the model and those obtained experimentally are within the coefficient of variation of the model, which shows that it accurately predicts the results obtained (Fig. S5).

Therefore, sample 6 shown in Table 1, was the best sample for the preparation of the almond and flaxseed drink. It had the highest mass fraction of omega-3 fatty acids (4.27 mg/g) and an acceptable viscosity value (6.03 mPa·s). We carried out the analyses to characterize the drink from this sample.

### Fatty acid composition

A total of 17 fatty acids were identified in both beverages, with the total sum of fatty acid mass fractions of 90.24 and 78.42 mg/g in the beverages with almonds and beverages with almond plus flaxseed, respectively (Table 3). The main difference between the two beverages was observed in the fatty acid 18:3n-3, where the addition of flaxseed resulted in a 1960-fold increase in mass fraction.

**Table 3:** Fatty acids mass fraction in plant-based almond drinks

Fatty acid	*w*(fatty acid)/(mg/g)		
	Almond		Almond and flaxseed
6:0	(0.40±0.01)ª	(0.08±0.01)^b^	
8:0	(0.19±0.02)ª	(0.04±0.01)^b^	
10:0	(1.10±0.03)ª	(0.08±0.01)^b^	
12:0	(0.08±0.00)^a^	(0.10±0.01)^b^	
14:0	(0.05±0.00)^a^	(0.09±0.01)^a^	
16:0	(5.57±0.08)ª	(4.7±0.2)^b^	
16:1	(0.55±0.01)ª	(0.56±0.04)^a^	
17:0	(0.04±0.00)^a^	(0.03±0.00)^a^	
17:1	(0.07±0.00)^a^	(0.04±0.00)^b^	
18:0	(1.14±0.01)ª	(1.05±0.01)^b^	
18:1n-9	(57.2±0.8)ª	(46.8±2.8)^b^	
18:2n-6	(18.5±0.2)ª	(15.9±1.0)ª	
18:3n-3	(0.20±0.02)^a^	(3.9±0.2)^a^	
20:1n-9	(0.05±0.00)^a^	(0.02±0.00)^b^	
22:0	(0.06±0.01)ª	(0.07±0.00)^a^	
24:0	(5.04±0.05)ª	(5.04±0.03)^a^	
Saturated	(13.7±0.4)ª	(11.2±0.4)^b^	
Monounsaturated	(57.9±3.8)ª	(47.4±3.4)^b^	
Polyunsaturated	(18.7±1.0)ª	(19.8±1.2)^a^	
Total	(90.2±3.1)ª	(78.4±3.0)^b^	

Results are expressed as mean value±standard deviation (S.D.), *N*=3. Values ​​with different letters in superscript in the same row are significantly different (p<0.05) according to Tukey's test

The significant increase is because flaxseed is a known source of unsaturated fatty acids, which account for more than 30 % of the total composition. Linolenic acid (18:3n-3) is the most abundant and makes up nearly half of the total fatty acids, followed by oleic acid (C18:1-n6) (24.33 %) and linoleic acid (C18:2-n9) (15 %) (*40*). In contrast, studies have shown that lipids make up more than 50 % of the total net mass of almonds, with a higher amount of oleic (79.9 %) and linoleic (6.7 %) fatty acids, and a low amount of saturated fatty acids (*41*). By adding flaxseed to the almond drink, it is possible to increase the content of alpha-linolenic acid (18:3n-3) in the drink (Table 3), while the content of alpha-linolenic acid (ALA) in the almond drink was 0.20 %. Thus, by replacing only 25 % of almonds with flaxseeds in the preparation of the almond drink to obtain a drink that is a source of omega-3 fatty acids of plant origin, we were able to increase the ALA mass fraction to 3.92 mg/g of the sample. This result therefore shows that we were able to enrich the plant-based almond drink with 1960 times more ALA, thus complying with the legislation on food enrichment with alpha-linolenic acid according to the Resolution of the Collegiate Board No. 28 of 26 July 2018 of ANVISA (*23*). This regulation states that food enriched with ALA must contain at least 0.24 g, which is the recommended amount of this fatty acid for a healthy adult. Similar fortification results were reported by Correa *et al*. (*42*).

The results in Table 3 show that there was no significant difference in the amount of palmitic acid (C16:0) between the two drinks. On the contrary, the mass fraction of fatty acids 18:1n-9 and 18:2n-6 decreased in the enriched drink, with oleic acid decreasing by around 10 % and linoleic acid by around 2 %, which is because flaxseed contains a lower amount of these fatty acids than almonds. Thus, when comparing the saturated, monounsaturated and polyunsaturated fatty acids between the almond drink and the enriched drink, we can see a decrease of 2, 10 and 1 % in Table 3. Considering the cardioprotective benefits and the additional mental and physical health benefits in humans, such as a lower risk of chronic diseases and cognitive decline associated with omega-3 fatty acid consumption (*43*), we can conclude that the fortification of the almond-based vegetable drink with omega-3 fatty acids from flaxseed is beneficial for vegetarians and vegans. These individuals need an adequate daily intake of ALA to ensure sufficient levels of EPA and DHA, as their consumption of omega-3-rich animal-based foods is low (*44*). In addition, this fortification can help reduce the cost of the beverage as flaxseed is more affordable than almonds.

### Centesimal composition

In the centesimal composition of the plant-based almond drinks in Table 4, it can be observed that the obtained moisture values (88.21 to 88.32 %) did not change with the addition of flaxseed, which was expected because the product is liquid and the moisture content of both seeds is similar. Similar moisture values were reported by Hussein *et al*. (*45*). Protein mass fractions ​​(2.37 to 2.29 %) does not change with the addition of flaxseed because flaxseed and almond seeds have the same protein content (about 20 %) in their composition according to studies by Kouamé *et al*. (*9*), In contrast, the addition of flaxseed led to a decrease in the lipid mass fraction (from 6.53 to 4.52 %), which is because flaxseed contains a lower lipid content than almonds. Therefore, when we reduced the mass of almonds in the preparation and replaced it with flaxseed, the lipid mass fraction decreased (*46*). Regarding the carbohydrate mass fraction, an increase (from 2.64 to 4.68 %) was observed in the plant-based almond and flaxseed drinks, which can be attributed to higher carbohydrate content in flaxseed than in almonds (*46*,*47*). No significant differences were observed in the ash mass fraction (0.25 to 0.19 %). The composition of carbohydrates, proteins and lipids in each drink influenced the caloric value (Table 4). Thus, the plant-based drink made from almonds and flaxseed had a lower caloric value (68.54 %) than the almond drink (83.81 %), which is favorable for the mixed drink, as it is not only a source of omega-3 fatty acids, but also an option for a balanced diet.

**Table 4:** Centesimal composition of plant-based beverages

Parameter	*w*/%		
	Almond		Almond and flaxseed
Humidity	(88.2±0.8)ª	(88.3±0.4)^a^	
Ash	(0.25±0.01)ª	(0.19±0.01)^b^	
Lipid	(6.530.9)ª	(4.5±0.3)^b^	
Protein	(2.4±0.1)ª	(2.3±0.2)^a^	
Carbohydrate	(2.6±0.2)^b^	(4.7±0.8)^a^	
*E*/kcal	(83.8±0.1)^a^	(68.5±1.6)^b^	

Results are expressed as mean value±S.D., *N*=3. Values ​​with different letters in superscript in the same row are significantly different (p<0.05) according to Tukey's test

### The analysis of pH value and soluble solids

The pH value and acidity are crucial factors in the assessment of food quality. The pH values of the almond and almond-flaxseed drinks are shown in Table 5. It was found that the addition of flaxseed did not affect the pH or acidity of the enriched drink, as the values remained constant. This finding is consistent with the results of Veena *et al.* (*47*), who reported no changes in pH or acidity when cow's milk was fortified with flaxseed to increase its omega-3 fatty acid content. However, a significant increase in soluble solids was observed in the almond-flaxseed drink, likely due to the contribution of additional soluble compounds from flaxseed, such as polysaccharides and soluble fiber.

**Table 5:** The analysis of pH value and soluble solids of plant-based beverages

Centesimal	Almond	Almond and flaxseed
pH	(6.43±0.02)^a^	(6.48±0.03)^a^
Soluble solids/°Brix	(7.1±0.00)^b^	(8.1±0.00)^a^
Titratable acidity/%	(0.02±0.00)^a^	(0.02±0.00)^a^

Results are expressed as mean value±S.D., *N*=3. Values ​​with different letters in the same row are significantly different (p<0.05) according to Tukey's test

### Colorimetric analysis

The results of the color parameters are shown in Table 6. A significant difference (p<0.05) was observed among the vegetable beverages, with the addition of flaxseed being the main factor responsible for these changes. When evaluating the L* parameter (luminosity), the samples were closer to 100, indicating a stronger tendency towards white. In the study by Zheng *et al.* (*48*), who developed an almond-based milk analog, the L* value was 71.4±0.1, which is similar to the values found in the present study (75.2±0.2 and 76.3±0.1) and shows comparable clarity.

**Table 6:** Color parameters of plant-based beverage formulations

**Color parameter**	**Almond**	**Almond and flaxseed**
L*	(75.2±0.2)^b^	(76.3±0.1)^a^
a*	(3.38±0.05)^b^	(3.95±0.05)ª
b*	(5.1±0.1)^b^	(6.87±0.03)^a^

Results are expressed as mean value±S.D., *N*=3. Values with different letters in superscript in the same row are significantly different (p<0.05)

For the parameters a* and b*, the values ​​found are positive (+), which shows a tendency towards red and yellow colors, justified by the presence of carotenoids in the raw materials used, with a stronger tendency of the beverage with added flaxseed, explained by the analysis of total carotenoids, in which the sample had a higher concentration than the almond beverage. The increase in the parameter a* was observed by Lima *et al.* (*49*), who added pre-emulsified flaxseed oil to beef sausages and found that the higher the concentration of flaxseed oil, the higher the value of a*.

### DPPH and carotenoid analysis

The 1,1-diphenyl-2-picrylhydrazyl (DPPH) radical scavenging method is the most commonly used to evaluate the antioxidant capacity of a product by spectrophotometric measurement of the decrease in the absorbance of the DPPH radical after the start of the reaction (*50*). Although not nutritionally essential, carotenoids serve as precursors of vitamin A and play a role in mitigating oxidative stress and free radical damage (*51*). Flaxseed is not only rich in fatty acids and fiber, but also contains a high concentration of phenolic acids, including chlorogenic acid, p-hydroxybenzoic acid, ferulic acid, vanillic acid and coumaric acid, which are among its primary bioactive compounds. It also contains lignans, such as matairesinol, pinoresinol, diphylline and secoisolariciresinol, which are present in smaller quantities. Flaxseed is also an excellent source of vitamins, including tocopherol and tocotrienols, as the most abundant forms of vitamin E, which are responsible for boosting its antioxidant effects (*52*).

The addition of flaxseed in foods therefore increases the antioxidant activity. In the optimized formulation of the almond and flaxseed drink, the DPPH and carotenoid values ​​increased considerably with the addition of flaxseed, from 6.54 μmol/g and 0.41 μg/100 g in the pure almond drink to 17.84 μmol/g and 1.81 μg/100 g in the drink mixed with flaxseed. These results show that the combined drink has a significantly higher antioxidant capacity than the pure almond drink, with a statistically significant difference (p<0.05). The inclusion of flaxseed in the formulation thus proves to be an effective strategy to increase the antioxidant effect of the drink.

Flaxseed has previously been used as a supplement to increase the nutritional value of food products. Additionally, studies have reported that supplementing with flaxseed increases omega-3 fatty acid content and thus improves the antioxidant potential of foods (*53*). A study in which soy and flaxseed were supplemented in a South African beverage called mahewu showed that these two ingredients contribute to antioxidant activity and phenolic content (*54*). Another example of flaxseed fortification was reported by Qin *et al*. (*55*) in the development of high yielding wheat bread. In that study, flaxseed bagasse was added, which is a byproduct of oil extraction and has high nutritional value. Thus, the bread produced did not lose its quality. The added flaxseed bagasse flour had a double effect on the gluten network of the wheat flour, which improved its yield. It also promoted antioxidant activity, glucose reduction capacity, water mobility, free water distribution of the bread and slowed down food degradation.

Although the increased antioxidant and carotenoid activity in the formulated beverage may help inhibit lipid oxidation of omega-3 fatty acids, the main cause of oil rancidity, this oxidation occurs through the reaction of oxygen with unsaturated fatty acids, which negatively affects the aroma, flavor and overall sensory quality of the beverage (*56*).

Future studies will be necessary to evaluate the effects of factors such as shelf life, storage temperature, heating, pasteurization, type of packaging, addition of natural or synthetic antioxidants and encapsulation of the oil on the oxidative stability of omega-3 fatty acids. Research in this area is essential for improving oxidative stability and extending the shelf life of the product.

## CONCLUSIONS

This study has successfully demonstrated the feasibility of optimizing a plant-based beverage made with almonds and flaxseeds, which are rich in alpha-linolenic acid. Using the D-optimal mixing method, the ideal formulation was determined, containing 15.426 g of almonds, 6.000 g of flaxseeds and 3.571 g of sweeteners (xylitol and erythritol). This combination not only significantly increased the omega-3 content, but also the antioxidant and carotenoid contents. With a remarkable 1960-fold increase in alpha-linolenic acid, the beverage proves to be a natural and cost-effective alternative. Additionally, the application of the response surface model proved effective in optimizing the desired formulation and emphasized the scientific importance of this research. It shows that an optimized formulation can positively affect the food industry by promoting healthier and more sustainable options. Therefore, this drink not only benefits the health of consumers, but also represents a significant advance in the availability of plant-based products on the market.
